# The metabolic pathway regulation in kidney injury and repair

**DOI:** 10.3389/fphys.2023.1344271

**Published:** 2024-01-12

**Authors:** Wenbin Tang, Qingqing Wei

**Affiliations:** ^1^ Health Management Center, Xiangya Hospital, Central South University, Changsha, Hunan, China; ^2^ Department of Cellular Biology and Anatomy, Medical College of Georgia, Augusta University, Augusta, GA, United States

**Keywords:** acute kidney injury, maladaptive repair, oxidative phosphorylation, glycolysis, fatty acid β-oxidation, pentose phosphate pathway, amino acids, ketone bodies

## Abstract

Kidney injury and repair are accompanied by significant disruptions in metabolic pathways, leading to renal cell dysfunction and further contributing to the progression of renal pathology. This review outlines the complex involvement of various energy production pathways in glucose, lipid, amino acid, and ketone body metabolism within the kidney. We provide a comprehensive summary of the aberrant regulation of these metabolic pathways in kidney injury and repair. After acute kidney injury (AKI), there is notable mitochondrial damage and oxygen/nutrient deprivation, leading to reduced activity in glycolysis and mitochondrial bioenergetics. Additionally, disruptions occur in the pentose phosphate pathway (PPP), amino acid metabolism, and the supply of ketone bodies. The subsequent kidney repair phase is characterized by a metabolic shift toward glycolysis, along with decreased fatty acid β-oxidation and continued disturbances in amino acid metabolism. Furthermore, the impact of metabolism dysfunction on renal cell injury, regeneration, and the development of renal fibrosis is analyzed. Finally, we discuss the potential therapeutic strategies by targeting renal metabolic regulation to ameliorate kidney injury and fibrosis and promote kidney repair.

## 1 Introduction

The kidney consumes a large amount of energy for its function to maintain body homeostasis by filtering blood, excreting waste products, and balancing electrolytes (Ronco et al., 2019; Singh, 2023). The energy metabolism in the kidney involves multiple pathways to actively generate ATPs and other metabolites essential for renal functions. Renal cells from different segments of the nephron may preferentially utilize one metabolic pathway over another, depending on their specific functions and energy requirements (Ronco et al., 2019). In normal kidneys, the energy sources primarily include glucose, fatty acids, amino acids, and ketone bodies (Gewin, 2021; Rojas-Morales et al., 2021).

Meanwhile, the kidney is an organ that is highly susceptible to acute injury, which can result from various risk factors such as ischemia, nephrotoxins, sepsis, or rhabdomyolysis (Zuk and Bonventre, 2016). During acute kidney injury (AKI), the renal cells, especially the proximal tubular cells, suffer from significant cell death associated with the deprivation of energy supply and/or dysregulation of energy metabolism, leading to acute loss of renal function (Basile et al., 2012). Following AKI, the kidney may undergo an adaptive repair to a complete recovery or a maladaptive repair to progress to chronic kidney disease (CKD). The maladaptive repair is featured by metabolic reprogramming in the kidney, shifting the major energy production from citric acid cycle/oxidative phosphorylation to glycolysis (Ferenbach and Bonventre, 2015; Wen et al., 2021). This metabolic reprogramming exerts divergent effects on different renal cells and further promotes tubular cell degeneration, inflammation, and fibrosis (Ding et al., 2017; Wei et al., 2019; Yang et al., 2023a; Xu et al., 2023). Overall, the dysregulation of metabolism in kidney injury and repair not only affects the kidney energy homeostasis through ATP production dysfunction but also influences various cellular functions via dual-functional enzymes and regulatory metabolites derived from different metabolic pathways.

In this review, we will introduce the typical energy metabolic pathways in the kidney and delve into the aberrant energy metabolism regulation in AKI and maladaptive repair. After exploring the distinct pathological roles of dysregulation of various metabolisms and the associated signaling pathways in renal cells, we will discuss the potential therapeutic strategies for AKI treatment and the prevention of its progression to CKD.

## 2 Bioenergetics in the normal kidney

### 2.1 Carbohydrate metabolism

With glucose as the foundational fuel to generate ATP, carbohydrate catabolism is the predominant energy production mechanism in most renal cells except proximal tubular cells (Gewin, 2021). The catabolic pathways include the oxidative phosphorylation and citric acid cycle and the glycolysis pathway. The pentose phosphate pathway (PPP) is an alternative anabolism pathway from glycolysis, which is critical for NADPH synthesis and oxidative stress suppression.

#### 2.1.1 Glycolysis

**FIGURE 1:**
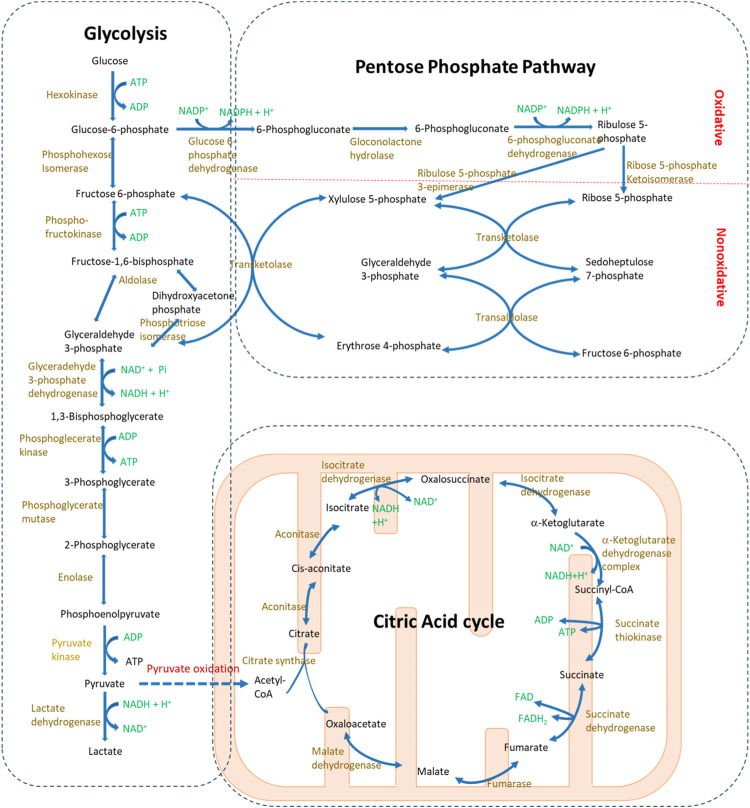
Glucose metabolism pathways in mammalian cells. Glucose serves as the fundamental substrate for bioenergy production, primarily through glycolysis and the citric acid cycle. During glycolysis, glucose is catabolized into pyruvate in the cytosol. Under anaerobic conditions, pyruvate is further converted to lactate. Conversely, in the presence of oxygen, pyruvate enters the mitochondria where it undergoes the citric acid cycle. Additionally, the pentose phosphate pathway, a branch metabolic pathway from glycolysis, is responsible for producing NADPH and supplying base metabolites for nucleotide synthesis.

Glycolysis is a central pathway in glucose catabolism and catalyzed by a series of enzymes located in the cytosol (Figure 1) (Janson and Tischler, 2018). Under aerobic conditions, this process yields pyruvate and ATP from glucose, while under anaerobic conditions, it results in the formation of lactate and ATP. In the presence of oxygen, the end product, pyruvate, can enter the mitochondria and be further converted to acetyl coenzyme A (acetyl-CoA), serving as a starting metabolite in the citric acid cycle. However, under anaerobic conditions, the accumulation of lactate may lead to acidosis.

Under normal conditions, the glycolysis level is not evenly distributed in the kidney. The inner medulla papilla and distal convoluted tubules are the major renal compartments showing high glycolytic activity (Ross et al., 1986). Furthermore, in the glomerulus, anaerobic glycolysis serves as the principal energy source for podocytes, playing a pivotal role in maintaining the function of the glomerular filtration barrier even with dysfunction of mitochondrial metabolism (Ozawa et al., 2015; Brinkkoetter et al., 2019). Although glycolysis levels are typically low in proximal tubular cells, a recent study indicates its crucial role in controlling phosphate homeostasis (Zhou et al., 2022a; Zhou et al., 2023). The blood phosphate can be sensed by proximal tubular cells, resulting in an increase in the glycolysis level. This process is characterized by enhanced activity of glyceraldehyde 3-phosphate dehydrogenase in the glycolysis pathway, which is coupled with the glycerol-3-phosphate dehydrogenase 1 activation through NAD/NADH balance modulation, leading to glycerol-3-phosphate (G-3-P) production. G-3-P is transported to the bone through blood, where it regulates the bone production of FGF23. This, in turn, provides feedback control over systemic phosphate levels by decreasing phosphate reabsorption in the proximal tubules and reducing intestinal phosphate uptake (Zhou et al., 2023).

#### 2.1.2 Citric acid cycle and oxidative phosphorylation

The citric acid cycle, in conjunction with oxidative phosphorylation, represents the most efficient bioenergetic pathway for ATP production to ensure the renal cell function (Janson and Tischler, 2018). The entire reaction takes place in mitochondria (Figure 1). Acetyl-CoA, derived from pyruvate, enters the cycle and reacts with oxaloacetate to form citrate. Following a series of 10 enzymatically catalyzed reactions, the cycle concludes with the regeneration of oxaloacetate. During oxidative phosphorylation, the respiratory chain complexes receive electrons from the products of the citric acid cycle, facilitating the transport of protons from the mitochondrial matrix to the intermembrane space. The proton gradient between the intermembrane space and matrix drives the ATP production catalyzed by ATP synthase. Of note, mitochondria also produce a significant amount of reactive oxygen species (ROS) during oxidative phosphorylation (Tirichen et al., 2021), which plays a crucial role not only in transducing cellular signals but also in inducing oxidative stress and cellular damage in renal diseases.

The kidney cells predominantly depend on mitochondrial bioenergetics to meet the substantial energy demands required for solute reabsorption functions (Bhargava and Schnellmann, 2017). Notably, both proximal tubular cells and distal convoluted tubular cells are abundant in mitochondria (McCormick and Ellison, 2015; Bhargava and Schnellmann, 2017). Proximal tubular cells, in particular, heavily rely on the citric acid cycle and oxidative phosphorylation for energy supply, owing to their low glycolytic activity. Thus, they are most susceptible to oxygen deprivation in pathological conditions. In addition, the presence of high glucose can especially suppress mitochondrial respiration through the Crabtree effect (Darshi et al., 2023).

#### 2.1.3 Pentose phosphate pathway

PPP is an anabolism pathway that branches from glycolysis (Figure 1) (Murray et al., 2012). Like glycolysis, the reactions of PPP occur in the cytosol; however, unlike glycolysis, PPP does not produce ATP. PPP can be divided into two phases: the irreversible oxidative phase and the reversible nonoxidative phase. The oxidative phase generates NADPH, which is essential for maintaining glutathione levels crucial for detoxification. The nonoxidative phase, on the other hand, produces ribose, vital for nucleotide and nucleic acid synthesis. PPP and glycolysis are intricately linked through shared metabolites, and alterations in the dynamics of one pathway inevitably influence the metabolic flux in the other.

### 2.2 Lipid metabolism

#### 2.2.1 Fatty acid β-oxidation

Fatty acid β-oxidation is a pivotal process in lipid metabolism that converts free fatty acids into acetyl-CoA, which then enters the citric acid cycle and undergoes oxidative phosphorylation (Murray et al., 2012). These free fatty acids are transported to the kidney via the bloodstream and must be activated prior to β-oxidation. The entire β-oxidation process is aerobic and takes place in the mitochondria. Furthermore, peroxisomes assist in breaking down very-long-chain fatty acids before they are oxidized in the mitochondria. Collectively, fatty acid β-oxidation, when followed by the citric acid cycle and oxidative phosphorylation, provides the highest ATP yield compared to other energy substrates. Hence, it is the most favored energy metabolism pathway for proximal tubular cells (Jang et al., 2020a; Gao and Chen, 2022).

#### 2.2.2 Ketone bodies

When fatty acid oxidation occurs at a high rate, ketone bodies are synthesized from acetyl-CoA by hepatocytes (Murray et al., 2012). These ketone bodies include acetoacetate, β-hydroxybutyrate, and acetone. Among them, acetoacetate and β-hydroxybutyrate are transported via the bloodstream to other organs as energy sources. Within the kidney, these ketone bodies are primarily reabsorbed by renal proximal tubular cells from the renal filtrate and are then oxidized into acetyl-CoA (Ferrier et al., 1992). The physiological concentration of ketone bodies supports kidney health during injury by aiding renal cells to survive from starvation conditions (Tajima et al., 2019; Rojas-Morales et al., 2021; Rojas-Morales et al., 2022; Liu and Yan, 2023). However, in pathological conditions such as diabetes, the overproduction of ketone bodies can lead to ketoacidosis, which is frequently associated with AKI (Orban et al., 2014; Huang et al., 2022).

### 2.3 Amino acid metabolism

Proteins undergo constant degradation and synthesis to sustain regular cellular functions. As the backbone of proteins, some amino acids are re-used during this protein turnover, while other extra amino acids undergo deamination. Their carbohydrate skeletons can either enter the citric acid cycle as substrates for energy production or be utilized to synthesis glucoses and fatty acids (Murray et al., 2012). The kidney plays a critical role in managing the amino acid reservoir for protein turnover by either absorbing or releasing specific amino acids such as glutamine, proline, serine, and cystine (Garibotto et al., 2010). It uptakes approximately 30% glutamine, 60% proline, 100% citrulline, 100% S-adenosylhomocysteine, and 90% cysteinylglycine from the bloodstream while releasing 100% of serine and cysteine, 50% arginine, 50% tyrosine, and 5%–20% lysine into the bloodstream. The nitrogen resultant from deamination will be converted into urea in the liver, which will be cleared out of the body by the kidney. Thus, the blood urea nitrogen level is an important index to monitor kidney function.

Among the twenty amino acids necessary for protein synthesis, nine are essential amino acids that cannot be synthesized by mammalian cells and must be acquired through diet (Murray et al., 2012). In recent years, the importance of maintaining a balance in the metabolism of branched-chain amino acids (BCAAs)—which include the three essential amino acids, namely, leucine, valine, and isoleucine—and aromatic amino acids (AAAs) (such as phenylalanine, tryptophan, tyrosine, and histidine, with three of them being essential) has gained attention in kidney injury and repair as emerging research studies show that their deficiency can cause malnutrition and progression of kidney diseases (Piret et al., 2021a; Barba et al., 2021; Mahbub et al., 2021; Shan et al., 2023). However, the specific roles and functions of these amino acids remain largely unexplored.

## 3 Metabolic dysregulation in AKI

AKI is characterized by a rapid decline in renal function. As a major renal disease, AKI is associated with high mortality in clinics. The leading causes for AKI encompass renal ischemia due to severe cardiovascular conditions or surgeries, nephrotoxicity resulting from toxins or medications (e.g., the chemotherapy drug cisplatin and myoglobin due to rhabdomyolysis), and sepsis. Although the pathophysiology of AKI varies due to its underlying causes, proximal tubular cells are usually identified as the primary site of injury. The lethal and sub-lethal injury of proximal tubular cells has attracted considerable research attention because of their essential role in reabsorption and their heavy reliance on energy production (Basile et al., 2012). Nevertheless, recent findings highlight the crucial role of other renal cells, including inflammatory and endothelial cells, in the progression of AKI (Zuk and Bonventre, 2016).

**FIGURE 2:**
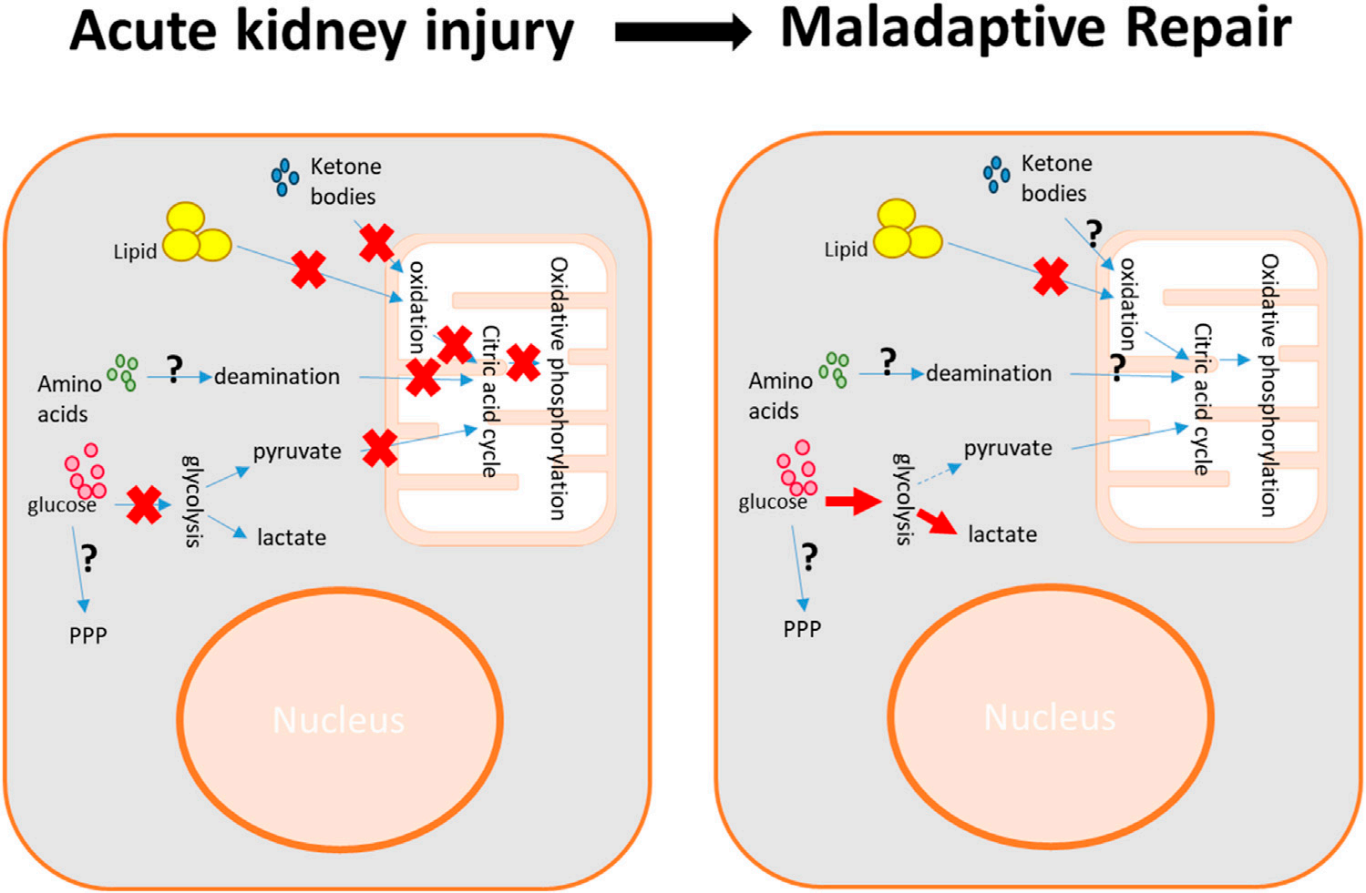
Metabolic impairment in kidney injury and reprogramming in kidney repair. During acute kidney injury, the deprivation of nutrients and oxygen results in a reduction in energy production, primarily marked by the suppression of glycolysis and mitochondrial bioenergetics. The maladaptive kidney repair is characterized by a shift in energy production from mitochondrial bioenergetics to glycolysis. The impairment of pathways such as the pentose phosphate pathway (PPP), amino acid metabolism, and ketone body metabolism varies depending on the specific pathological conditions.

In recent years, emerging studies have underscored the significance of metabolic dysregulation in the pathophysiology of AKI, which has been explored in comprehensive studies using systematic metabolomic profiling with either mass spectrometry or NMR spectroscopy (Portilla et al., 2006; Wei et al., 2014; Jouret et al., 2016; Huang et al., 2018; Ping et al., 2019; Standage et al., 2021; Davidson et al., 2022; Lim et al., 2023). These studies, undertaken by our team and a few other research groups, consistently highlight mitochondrial dysfunction as a common feature in different AKI models. The mitochondrial dysfunction leads to suppression of fatty acid β-oxidation, the citric acid cycle, and oxidative phosphorylation (Figure 2). In ischemia/reperfusion (I/R)-induced AKI, a disturbance in glycolysis is evident, due to both the insufficient glucose supply from the bloodstream and impaired gluconeogenesis in kidney proximal tubules (Wei et al., 2014; Legouis et al., 2020; Scantlebery et al., 2021) (Figure 2). However, PPP regulation is complicated depending on the original insults (Smith et al., 2014; Bushau-Sprinkle et al., 2020; Scantlebery et al., 2021). Additionally, dysregulation in inflammation-related tryptophan catabolism, osmolality, and impaired purine metabolism have also been reported in ischemic AKI (Wei et al., 2014; Jouret et al., 2016; Huang et al., 2018; Davidson et al., 2022). In sepsis-induced AKI, notable dysregulation in the metabolism of BCAAs has been observed (Standage et al., 2021). The specific roles and impacts of these energy production pathways in AKI have been the subject of extensive research studies and are summarized below.

### 3.1 Suppressed mitochondrial energy production

During AKI, the renal tubular cells, especially the proximal tubular cells, suffer from severe mitochondrial damage, associated with the loss of some major enzymes for mitochondrial bioenergetics (Jin et al., 2021). Furthermore, the renal vascular impairment, resulting from the endothelial dysfunction, adversely affects the oxygen delivery efficiency and, consequently, the mitochondrial function (Zuk and Bonventre, 2016). Thus, the energy production via fatty acid β-oxidation, the citric acid cycle, and oxidative phosphorylation is significantly suppressed. Given that the proximal tubule is the major renal compartment with high-energy production demand from mitochondria, in AKI resulting from various injurious factors, the accumulation of lipid droplets in the kidney has been commonly observed (Iwaki et al., 2019; Jang et al., 2020b; Bugarski et al., 2021; Xu et al., 2022; Yang et al., 2023b; Chen et al., 2023; Wang et al., 2023). Therefore, promoting mitochondrial biogenesis has been considered a major therapeutic strategy for AKI treatment (Clark and Parikh, 2020; Pabla and Bajwa, 2022).

Peroxisome proliferator-activated receptors (PPARs) are ligand-activated transcription factors that regulate the gene expression related to lipid metabolism and are crucial in regulating mitochondrial biogenesis (Gao and Gu, 2022). The activation of PPARs promotes fatty acid oxidation, enhancing the mitochondrial bioenergy production. The function of various PPAR isoforms has been extensively investigated in AKI. In ischemic AKI, the overexpression of PPARα has been shown to protect mice from renal injury (Li et al., 2009). In addition, PPARα prevents sepsis-induced AKI by promoting fatty acid metabolism, which in turn helps suppress inflammation (Iwaki et al., 2019). Both Krüppel-like factor 15 and ERK1/2 have been reported to regulate the transcription of PPARα (Collier et al., 2016; Piret et al., 2021b). In cisplatin-induced AKI, PPARα translocates from the nucleus to mitochondria, binding to cyclophilin D, which further suppresses its transcription activity and reduces fatty acid metabolism (Jang et al., 2020a). Another isoform PPARγ shows similar fatty acid metabolism regulation function, and its agonists have been reported to exert protective effects in experimental AKI models (Doi et al., 2007; Reel et al., 2013; Zhang et al., 2016; Chen et al., 2017; Singh et al., 2019; Liu et al., 2020; Sharma and Patial, 2022). Furthermore, PPARG coactivator 1 alpha (PGC1α) is a co-activator of PPAR isoforms and a critical regulator in mitochondrial biogenesis (Lynch et al., 2018). PGC1α is suppressed in AKI (Portilla et al., 2002; Tran et al., 2011) and its suppression or deficiency worsens the renal injury, while its induction in renal tubular cells ameliorates ischemic AKI (Tran et al., 2011; Ruiz-Andres et al., 2016; Tran et al., 2016; Fontecha-Barriuso et al., 2019). Despite the central role of the PPAR/PGC1α pathway in AKI, a recent clinical study of the PPARδ agonist ASP1128 did not show significant renal beneficial effects on reducing AKI incidence or severity after major cardiac surgery as expected (van Till et al., 2023). Overall, their therapeutic potential and effectiveness in clinical settings may vary, indicating a need for further development and research of new activating chemicals.

Since the depletion of functional mitochondria is a major pathological event in AKI, in recent years, the transplantation of mitochondria to scavenge the renal cells has been examined in AKI therapy (Doulamis et al., 2020; Jabbari et al., 2020; Pabla and Bajwa, 2022; Rossi et al., 2023). Initial studies by two independent research groups have demonstrated that mitochondrial delivery can attenuate ischemic AKI in both rat and swine models (Doulamis et al., 2020; Jabbari et al., 2020; Rossi et al., 2023). No significant safety issue has been detected by mitochondrial transplantation (Doulamis et al., 2020). However, there are still challenges to be addressed. Ensuring the viability of the transplanted mitochondria remains a significant concern, as their functionality is crucial for the therapeutic benefit. Additionally, the efficacy of mitochondrial transplantation in AKI scenarios other than I/R is yet to be established.

Notably, hypoxia, another key pathological condition in AKI, limits the mitochondrial bioenergetics even in the presence of functional mitochondria. Paradoxically, excessive oxygen consumption by mitochondria may further aggravate renal hypoxia. Thus, it raises a question whether the kidney can be rescued simply by increasing mitochondrial bioenergetics. Kidney oxygen consumption is directly proportional to the glomerular filtration rate and sodium reabsorption (Redfors et al., 2010). Zhou (2023) highlights an alternative strategy for AKI treatment: reducing renal oxygen consumption by inhibiting sodium reabsorption in renal tubular cells. While this strategy has shown promise in animal models, its clinical effectiveness remains controversial. Thus, it underscores further investigation to elucidate how to maintain a balance of renal oxygen delivery, mitochondrial function, and oxygen consumption during AKI therapy.

### 3.2 Disturbed glycolysis

Although the total glycolysis level is suppressed in AKI, the mitochondrial dysfunction, especially in proximal tubules, shifts the balance of energy production toward glycolysis. However, the pathological function of glycolysis is complicated due to its close connection with other metabolic pathways (Figure 1), and its impact varies across different renal cells (renal tubular cells vs. inflammatory cells) and AKI conditions. Glycolysis can be beneficial as it helps prevent ATP depletion in renal tubular cells. Meanwhile, the suppression of glycolysis may shift the energy production to other pathways such as citric acid cycle and PPP. For instance, preconditioning treatments with meclizine, enarodustat, or AMPK activators, which enhance glycolysis in proximal tubular cells, have been shown to ameliorate ischemic AKI both *in vitro* and *in vivo* (Kishi et al., 2015; Lieberthal et al., 2016; Ito et al., 2020). Additionally, dichloroacetate treatment or knockout of its target pyruvate dehydrogenase kinase 4, which shifts metabolism from glycolysis to the citric acid cycle and oxidative phosphorylation, has been effective in protecting mice from cisplatin-induced nephropathy (Galgamuwa et al., 2016; Oh et al., 2017). In contrast, in AKI induced by sepsis, the inhibition of glycolysis with agents like 2-deoxyglucose has been observed to have a protective effect in mice (Ji et al., 2021; Tan et al., 2021). One possibility is that glycolysis may enhance inflammation to promote M1 macrophage polarization (Takakura and Zandi-Nejad, 2019; Yang et al., 2023c). Intriguingly, AMPK activation still shows protection in sepsis-induced AKI, possibly due to its role in enhancing oxidative phosphorylation as well (Jin et al., 2020). Furthermore, tubular-specific knockout of pyruvate kinase M2 (PKM2), a key enzyme in glycolysis, has been reported to protect ischemic AKI by switching the metabolism to PPP (Zhou et al., 2019).

Disturbances in glycolysis during AKI result in altered levels of glycolytic metabolites, which can further influence renal injury and recovery. First, the acute loss of renal function leads to lactate accumulation, and the elevated serum lactate levels have been considered an index of AKI severity (Zhou et al., 2022b). One potential pathological role of lactate in AKI is to downregulate SIRT3 and p-AMPK, followed by autophagy inhibition (Tan et al., 2021). Furthermore, lactic acidosis has been considered to drive the development of CKD (Wesson et al., 2020). Pyruvate, the end product of anaerobic glycolysis and the initial metabolite for the citric acid cycle (Figure 1), decreases in the injured kidney (Zager et al., 2014). The renal protective effects of pyruvate have been identified in various AKI conditions, where it helps reduce oxidative stress and suppress inflammation (Salahudeen et al., 1991; Leelahavanichkul et al., 2008; Jun et al., 2018). In addition, fructose-1,6-bisphosphate, an intermediate metabolite in the glycolysis pathway (Figure 1), has been shown to mitigate kidney injury from I/R or cisplatin nephrotoxicity, although the underlying mechanism is not fully understood (Antunes et al., 2006; Azambuja et al., 2011).

Finally, some multifunctional enzymes in the glycolysis pathway, such as PKM2 and 6-phosphofructo-2-kinase/fructose-2,6-biphosphatase 3 (PFKFB3), play crucial roles in regulating kidney injury through mechanisms beyond their primary metabolic functions. PKM2 is a rate-limiting enzyme to produce pyruvate in glycolysis. The specific knockout of PKM2 in renal tubular cells has been shown to protect mice from ischemic AKI and cisplatin-induced nephropathy (Zhou et al., 2019; Xie et al., 2023). While the exact mechanism through which PKM2 regulates ischemic AKI is not fully understood, its knockout seems to reduce oxidative stress and enhance PPP. In the kidneys injured by cisplatin, PKM2 is phosphorylated and translocates to mitochondria, leading to mitochondrial fragmentation and exacerbating tubular injury (Xie et al., 2023). PFKFB3, another enzyme in the glycolysis pathway, catalyzes the production of fructose 2,6-bisphosphate (F2,6P2), which in turn activates phosphofructokinase-1, a rate-limiting enzyme in glycolysis. PFKFB3 is significantly upregulated in the kidneys damaged by cisplatin, and its renal tubular-specific knockout or inhibition attenuates cisplatin-induced AKI (Wen et al., 2023). However, the detrimental role of PFKFB3 in kidney injury relies on the activation of CDK4 and the regulation of the cell cycle, rather than its metabolic activity. Considering the complexity of glycolysis-related metabolites and enzymes in different renal cells, much research is needed to examine the detailed function and mechanism of specific inhibitors or activators for glycolysis.

### 3.3 Dysregulation of PPP

The activity of PPP in AKI varies depending on the initial insults of the kidney. In ischemic AKI, there is an increase in PPP-related gene expression 24 h post-injury (Scantlebery et al., 2021). In sepsis-induced AKI, the activity of glucose-6-phosphate dehydrogenase (G6PDH), a key enzyme in PPP, is significantly elevated (Smith et al., 2014). In the case of cisplatin-induced AKI, despite a decrease in intermediate metabolites, G6PDH activity is induced (Bushau-Sprinkle et al., 2020). PPP is unique in its ability to produce NADPH, which is critical for controlling oxidative stress and protection against kidney injury (Weng et al., 2018). The deficiency of G6PDH is directly associated with AKI in clinics (Owusu et al., 1972; Abdel Hakeem et al., 2016; Talwar et al., 2019). Furthermore, the induction of G6PDH or PPP activity has been shown to protect the kidneys from cisplatin- or I/R-induced AKI (Zhou et al., 2019; Bushau-Sprinkle et al., 2020). Notably, glucose can shuttle between PPP and glycolysis. Therefore, both the activation of hexokinase and the knockout of PKM2 have been reported to enhance PPP activity in the kidney (Smith et al., 2014; Zhou et al., 2019). However, whether the activation of PPP can regulate glycolysis is unclear. In addition, because PPP not only benefits the detoxification of oxidative stress but also provides basic metabolites for nucleotide synthesis, the detailed mechanism of PPP in AKI progression needs further exploration.

### 3.4 Impaired amino acid metabolism

Various types of kidney injury lead to disturbances in amino acid metabolism, exhibiting different patterns depending on the nature of the injury. In ischemic AKI, there is a notable decrease in multiple amino acids, including glutamate, glutamine, tyrosine, proline, and methionine (Wei et al., 2014). Shan et al. (2023) have observed a significant reduction in plasma isoleucine levels following kidney I/R. In sepsis-induced AKI, a decrease in metabolites involved in BCAA metabolism has been detected (Standage et al., 2021). Following cisplatin treatment in mice, the urinary levels of alanine, leucine, and methionine have been significantly elevated, although the changes in their kidney levels have not been determined (Lim et al., 2023).

Our current understanding of the specific roles of individual amino acids in AKI is still evolving. Glycine has been extensively studied across various AKI conditions. Its administration has been shown to reduce free radical production and protect renal epithelial cells in both *in vitro* and *in vivo* models of ischemic AKI, as well as in lead- or cisplatin-induced nephrotoxicity (Heyman et al., 1991; Paller and Patten, 1992; Weinberg, 1992; Yin et al., 2002; Shafiekhani et al., 2019). However, this protective effect appears limited in milder injury conditions or chronic ischemia (Yin et al., 2002). Contrarily, Arora et al. (2014) have found that glycine administration can exacerbate ischemic AKI by activating the NMDA receptor. Moreover, glutamine supplementation has demonstrated universal protective effects in diverse AKI scenarios, including ischemic AKI, sepsis-induced AKI, cisplatin nephrotoxicity, and gentamycin-induced nephrotoxicity (Hu et al., 2012; Kim et al., 2015; Thomas et al., 2022; Zhan et al., 2022). L-Arginine deficiency has been identified in the kidney transplant recipients, and its supplementation protects rats from uranyl nitrate-induced AKI (Schramm et al., 2002). While BCAA metabolism has been reported to reduce aristolochic acid-induced kidney injury, potentially regulated by Krüppel-like factor 6 (Piret et al., 2021a), the mechanisms underlying these effects remain unclear. Overall, these findings underscore the complex and varied roles of amino acids in AKI, pointing to the need for much research to fully understand their functions and therapeutic potential in different AKI contexts.

### 3.5 Perturbed energy supply from ketone bodies

While the overproduction of ketone bodies can lead to ketoacidosis in diabetic conditions, increasing the risk of AKI (Orban et al., 2014; Huang et al., 2022), the enhancement of plasma levels of ketone bodies within the normal range protects renal cells from AKI injury (Tajima et al., 2019; Rojas-Morales et al., 2022; Gui et al., 2023). In a recent study by Gui et al. (2023), calponin 2 has been found to increase in AKI resulting from I/R or cisplatin nephrotoxicity. The knockdown of calponin 2 attenuates kidney injury by upregulating hmgcs2, the key enzyme in ketogenesis, and increasing β-hydroxybutyrate levels in mice (Gui et al., 2023). In addition, both ketogenic diet and β-hydroxybutyrate administration have demonstrated efficacy in ameliorating I/R-induced kidney injury (Tajima et al., 2019; Rojas-Morales et al., 2022). Notably, ketone bodies may offer renal protection through mechanisms beyond just energy production. In the study by Tajima et al. (2019), β-hydroxybutyrate can restore the histone acetylation of the FOXO3 promoter, thereby suppressing pyroptosis through the induction of FOXO3 expression. All these findings highlight the needs of further exploration of the metabolic- and non-metabolic-related mechanisms of ketone bodies in AKI.

## 4 Metabolic reprogramming in kidney repair

Following the acute injury phase, the kidney initiates self-repair processes to restore renal function. Clinically, many patients experience adaptive kidney repair with complete functional recovery within 1 week to 3 months, although the recovery rates can vary widely, ranging from 33% to 90% (Forni et al., 2017). However, a subset of patients may undergo maladaptive kidney repair, failing to achieve full functional restoration and progressing to CKD. As per the recent criteria proposed by the Acute Dialysis Quality Initiative (ADQI), patients who do not recover within 90 days are considered to have CKD (Forni et al., 2017). The kidney repair process involves multiple renal cell types, including the restoration of the renal vascular system through endothelial cell repair and regeneration, the re-establishment of functioning nephrons via renal tubular cell proliferation, and the modulation of the repair process by infiltrating and proliferating inflammatory cells (Ferenbach and Bonventre, 2015). In maladaptive repair, the disruption or renal vascular system results in hypoxia, nutrition deprivation, and oxidative stress accumulation, leading to cell cycle arrest in proliferating renal tubular cells. Concurrently, the infiltration of inflammatory cells may release more cytokines, which not only inhibit the renal cell repair but also promote myofibroblast activation and fibrosis development.

The disruption of kidney metabolism during the acute injury phase not only impacts the immediate functioning of the kidney but also influences the subsequent repair process. Meanwhile, kidney repair is featured by metabolic reprogramming, which regulates tubular degeneration, proliferation, and differentiation. This reprogramming includes the suppression of fatty acid β-oxidation, citric acid cycle, and oxidative phosphorylation, along with the induction of glycolysis. Although an increase in PPP has been observed in diabetic nephropathy, its rate-limiting enzyme G6PDH has been reported to decrease (Steer et al., 1985; Xu et al., 2005). Furthermore, the specific role of PPP activity and its associated enzymes in other kidney repair and fibrosis conditions remains unclear. Meanwhile, emerging studies are highlighting the critical role of amino acid metabolism in the kidney repair process (Kumar et al., 2012; Pillai et al., 2019; Ikeda, 2020; Barba et al., 2021; Lanzon et al., 2021; Prasad et al., 2023). Finally, the information of ketone bodies and glycogen in kidney repair is lacking, although the ketogenic diet has been reported to regulate fatty acid β-oxidation and suppress renal fibrosis (Qiu et al., 2023).

### 4.1 Metabolic switch to glycolysis in kidney repair

Metabolic reprogramming, particularly the shift in energy production from the citric acid cycle and oxidative phosphorylation to glycolysis, is a key pathological hallmark of maladaptive kidney repair (Schaub et al., 2021) (Figure 2). The upregulation of glycolysis has long been recognized in the repair and regeneration of proximal tubular cells following ischemic injury. This includes increased production of glycolytic end products and elevated levels of key glycolytic enzymes such as hexokinase 2 (HK2), 6-phosphofructo-2-kinase/fructose-2,6-bisphosphatase 3 (PFKFB3), and pyruvate kinase M2 (PKM2) (Lan et al., 2016). This metabolic shift was further validated by a recent study by Wang et al. (2022), which used high spatial resolution measurements to examine proximal tubular metabolism *in situ* within the kidney. In the same kidney with ischemic injury, the maladaptive repaired proximal tubules had significantly more lactate accumulation and less citric acid cycle activity compared to those repaired or healthy proximal tubules. Notably, the S3 segment of proximal tubules showed even greater lactate buildup compared to S1/S2 segments. An intriguing observation was that, compared to uninjured proximal tubules in the sham-operated kidneys, the healthy proximal tubules in the repaired kidneys still displayed higher lactate levels and lower concentrations of citric acid cycle metabolites, indicating some degree of prolonged metabolic reprogramming in the kidneys that have undergone repair.

The role of enhanced glycolysis in pathology has been a subject of intense study in recent years, yielding controversial results. In research using zebrafish models to investigate the energy metabolism, CXCL12 and MYC have been pinpointed to promote renal repair through the upregulation of glycolysis (Yakulov et al., 2018). However, the subsequent validation experiments using mouse models with CXCL12 or MYC knockout in renal tubules have failed to distinguish the renal injury and repair phase, leaving the conclusion obscure. In another study utilizing a PFKFB2 mutant knock-in mouse model, glycolysis has been suppressed, yet renal fibrosis has markedly increased, following ureteral obstruction or folic acid-induced injury (Lee et al., 2020). Conversely, data from our research and those of others have indicated that inhibiting glycolysis can significantly enhance renal repair and ameliorate renal fibrosis (Ding et al., 2017; Wei et al., 2019; Shen et al., 2020; Ye et al., 2021; Yang et al., 2023c; Yang et al., 2023d; Xu et al., 2023). Specifically, glycolysis inhibition can reduce macrophage infiltration and differentiation, fibroblast activation and proliferation, and the pericyte–fibroblast transition (Shen et al., 2020; Ye et al., 2021; Yang et al., 2023c; Yang et al., 2023d; Xu et al., 2023). The role of glycolysis in renal tubules is complicated. We have found that glycolysis inhibitors did not suppress the partial epithelial–mesenchymal transition in cultured proximal tubular cells but decreased renal tubular cell apoptosis in the obstructed kidneys (Wei et al., 2019). It is possible that the differentially injured proximal tubular cells may further regulate renal inflammation and fibroblast activation (Shen et al., 2020; Yang et al., 2023d; Kayhan et al., 2023). Overall, glycolysis inhibition may have divergent effects on different renal cells.

### 4.2 Fatty acid β-oxidation suppression in kidney repair

The increase in glycolysis levels in repaired kidneys is always associated with the dysregulation of lipid metabolism (Harzandi et al., 2021). This is particularly evident in proximal tubules, which rely heavily on fatty acid β-oxidation for energy production and contain abundant peroxisomes and mitochondria. These cells are especially susceptible to mitochondria damage and lipid metabolism dysregulation after kidney injury, resulting in lipid accumulation in the kidney and the presence of fatty acids in urine (Bataille et al., 2018; Jang et al., 2020a; Dhillon et al., 2021; Gewin, 2021; Gu et al., 2022; Rinaldi et al., 2022). Genome-wide transcript profiling has identified various patterns of aberrant expression of fatty acid metabolism regulators in proximal tubular cells across different types of fibrotic kidneys after ischemia/reperfusion, folic acid injury, or kidney transplant (Bataille et al., 2018; Dhillon et al., 2021; Rinaldi et al., 2022). In the folic acid-treated kidneys, estrogen-related receptor alpha, a nuclear receptor pivotal in regulating fatty acid oxidation, has been reported to promote proximal tubular cell lipid metabolism and differentiation (Dhillon et al., 2021). In I/R-induced kidney injury, αKlotho deficiency-induced lipid droplet accumulation in the kidney is associated with CKD transition (Wang et al., 2023). In addition, protein phosphatase 2Acα, which can inhibit fatty acid β-oxidation and simultaneously enhance glycolysis through the dephosphorylation of phospho-acetyl-CoA, is induced in the fibrotic kidneys (Gu et al., 2022). This enzyme induction suppresses kidney repair by increasing tubular cell death and fibrosis in the obstructed kidney. The enhanced fatty acid β-oxidation by PGC1α activator also inhibits the pericyte–myofibroblast transition to prevent AKI–CKD transition (Xu et al., 2023). However, our current understanding of the roles of these fatty acid metabolism-related genes in kidney repair remains incomplete. Additionally, how peroxisomes participate in this lipid metabolism regulation is unclear.

### 4.3 Amino acid homeostasis in kidney repair

Amino acids, serving as fundamental components for protein synthesis, play a crucial role in kidney repair. Essential amino acids are transported to the kidney via the bloodstream and are absorbed by kidney cells through amino acid transporters (Broer, 2008). However, studies examining plasma concentrations of amino acids have yielded controversial results (Bednarek-Skublewska et al., 2002; Kumar et al., 2012; Ikeda, 2020; Lanzon et al., 2021; Mahbub et al., 2021). While most studies report a decrease in BCAAs in plasma (Bednarek-Skublewska et al., 2002; Kumar et al., 2012; Ikeda, 2020), Mahbub et al. (2021) have observed increased BCAAs in the blood of CKD patients, correlating strongly with a decline in the estimated glomerular filtration rate. One possible reason is that dialysis patients might experience nutritional loss during the treatment process (Ikeda, 2020). It is noteworthy that change patterns of BCAA and AAA are different (Mahbub et al., 2021). In a recent study by Pillai et al. (2019), the diet supplement effect of BCAA or AAA has been examined in the 5/6 nephrectomy rat model. Intriguingly, BCAA and AAA supplements have shown divergent impacts on kidney injury and fibrosis development. BCAA supplements promote fibrosis, while AAA supplements are protective for the kidneys. Conversely, another animal study has reported conflicting findings regarding the role of AAAs, indicating that a low AAA diet protects rats from adenine-induced nephrotoxicity, reducing proteinuria, fibrosis, and inflammation (Barba et al., 2021). Overall, our understanding of amino acid homeostasis in kidney repair remains quite limited.

## 5 Conclusion and perspectives

In conclusion, the complex interplay of metabolic pathways plays a critical role in kidney injury and repair. The acute injury disturbs different metabolic pathways in the kidney due to mitochondrial damage, lack of nutrition, and oxygen deprivation. This metabolic dysfunction promotes kidney injury not only through energy and ATP depletion but also impacting various signaling pathways through different metabolites and multifunctional enzymes. Furthermore, it also affects the metabolic reprogramming in the kidney repair phase, leading to aberrant upregulation of glycolysis. Importantly, the therapeutic potential of targeting these metabolic pathways, ranging from mitochondrial bioenergetic production to modulation of glycolysis, PPP activity, and amino acid metabolism, opens new avenues for intervention. However, the differential responses in various kidney injury models highlight the necessity for exploration of injury-specific therapeutic approaches. Moreover, a deeper understanding of the underlying mechanism of metabolic dysregulation, including the roles of key enzymes and metabolites, is crucial. Future research focusing on unraveling the intricate molecular mechanisms and identifying novel therapeutic targets can effectively advance the treatment of AKI and prevent the transition from AKI to CKD.
